# Impact of temperature and relative humidity variations on coda waves in concrete

**DOI:** 10.1038/s41598-024-69564-4

**Published:** 2024-08-14

**Authors:** Fabian Diewald, Marine Denolle, Jithender J. Timothy, Christoph Gehlen

**Affiliations:** 1 Centre for Building Materials (cbm), Technical University of Munich, 81245 Munich, Germany; 2 Department of Earth and Space Sciences, University of Washington, Seattle, WA 98195 USA

**Keywords:** Coda wave interferometry, Quality factor, Ultrasound, Monitoring, Temperature, Relative humidity, Concrete, Model, Non-destructive testing, Civil engineering, Civil engineering, Materials science

## Abstract

The microstructure of concrete can be affected by many factors, from non-destructive environmental factors through to destructive damage induced by transient stresses. Coda wave interferometry is a technique that is sensitive enough to detect weak changes within concrete by evaluating the ultrasonic signal perturbation compared to a reference state. As concrete microstructure is sensitive to many factors, it is important to separate their contributions to the observables. In this study, we characterize the relationships between the concrete elastic and inelastic properties, and temperature and relative humidity. We confirm previous theoretical studies that found a linear relationship between temperature changes and velocity variation of the ultrasonic waves for a given concrete mix, and provide scaling factors per Kelvin for multiple settings. We also confirm an anti-correlation with relative humidity using long-term conditioning. Furthermore, we explore beyond the existing studies to establish the relationship linking humidity and temperature changes to ultrasonic wave attenuation.

## Introduction

Today, large parts of the built environment are made from structural concrete. The safety of these structures is, in most cases, secured during regular, but often only annual, inspections rather than using continuous monitoring. Thus, these maintenance strategies rely on information from an ideally full update of the structures’ integrity on discrete inspection dates. In contrast, monitoring techniques have the advantage of continuously detecting structural changes and, therefore, allow preventive action to be initiated at an early stage before critical damage occurs. One recent and promising technique for monitoring heterogeneous media such as concrete is Coda Wave Interferometry (CWI)^1–3^. CWI leverages the fact that scattered waves propagate multiple times in perturbed media, summing the perturbations and changing the arrival times of waves relative to a reference state.

Multiple laboratory-scale studies have shown the technique’s potential to monitor microstructural changes in concrete as a consequence of external mechanical loads^4–7^ or due to temperature changes^8,9^. Furthermore, time-dependent concrete-specific phenomena can be characterized using CWI, e.g., the alkali-silica reaction^10^ or freeze-thaw damage^11^. Naturally, some of these investigations have been extended to large-scale concrete structures^12–16^, where CWI’s potential has been proven under controlled environmental conditions. Besides its high sensitivity to even weak changes in the material, one of the biggest challenges for CWI today is the simultaneous occurrence of condition changes that cause superimposed terms contributing to the velocity variation. The separation of these terms is often difficult under practical conditions because changes in environmental conditions can occur simultaneously (e.g., winters are wetter and colder in the mid-latitudes). This applies especially to interpreting the effect of transient changes, e.g., due to stresses or damage, because environmental temperature or humidity changes may induce a bias in measuring the ultrasonic signals’ phase lag^17–20^. In on-site applications, one specific challenge arises from the time-limited evaluation period (often only hours) because this is required so that only one parameter is changing^21^. However, to link the impact of temperature and damage, nonlinear ultrasonic techniques have been suggested either to link the influence of damage on ultrasonic waves using thermal modulation^22^ or to compensate for temperature effects using a thermal bias control by comparing the temperature-induced perturbation of test and reference specimens^23,24^. In our study, we address the necessity of separating the superimposed effects of environmental conditions affecting every concrete structure, i.e., temperature and relative humidity changes, on the speed at which ultrasonic waves propagate. We detected the changes in the coda waves using long-term, equilibrium, experiments and remained in the non-destructive regime to avoid having to address any additional impact of damage on the velocity variation. We separated the individual effects of temperature and relative humidity from each other and proposed a third-order polynomial regression model for their combined effects to predict velocity variations. The specimens are a normal concrete mix^25,26^. In addition to elastic wavespeed perturbations, monitoring the scattering properties of the materials, by measurements of amplitude decay in the coda waves, may be useful because the amplitude decay is particularly sensitive to damage. Therefore, we also demonstrate the changes in anelastic seismic properties (attenuation and scattering) in the seismic quality factor *Q*^27,28^ for both temperature and relative humidity variations.

## Materials and methods

### Coda wave interferometry

Coda wave interferometry is based on comparing deterministic and reproducible ultrasonic waveforms propagating in a heterogeneous medium along multiply scattered paths^21^. Any change in the medium’s scattering properties causes a perturbation of the waveform compared to the original, unperturbed waveform. Even weak changes can accumulate over many repeated wave paths between a sender-receiver pair and can be detected in late coda waves through phase differences, which can be expressed as a velocity perturbation *dv*/*v* of the ultrasonic signal, i.e., the velocity variation. The velocity variation is deduced from a linear regression of the time delay, which increases over signal time *t*, and the measurements of phase differences, *dt*. It is, therefore, most significant when measured in late windows that record scattered waves called the ultrasonic coda.

Multiple methods for CWI analysis have recently been proposed^2,29–31^, based on deriving a stretching factor $\varepsilon =dt/t=-dv/v$ from the calculation of a maximum signal correlation coefficient *CC* between a reference signal *u*(*t*) and a perturbed signal $\tilde{u}(t)$. Here, $t_1$ and $t_2$ mark the time window in which the velocity change is derived. In this study, we use the stretching technique^29^ to determine relative velocity changes *dv*/*v* in the medium according to Eq. (1).

1 $$\begin{aligned} CC(\varepsilon ) = \frac{\int _{t_1}^{t_2} u(t) \tilde{u}(t (1-\varepsilon ))\,dt}{\sqrt{\int _{t_1}^{t_2} u^2(t)\,dt \int _{t_1}^{t_2} \tilde{u}^2(t(1-\varepsilon ))\,dt}} \end{aligned}$$

The ultrasonic signals were preprocessed and were effectively noise-free. In the preprocessing procedure^32^, the value of $CC(\varepsilon )$ generally depends on the signal-to-noise ratio and the evaluated time-window. The cross-correlation coefficient and velocity variation were computed across the entire duration of each experiment using a window length starting with a pretrigger of 100 samples to the signal onset time and a total length of 2500 samples, equal to 2.5 ms.

### Quality factor

The quality factor *Q* describes the fractional loss of energy of an ultrasonic signal arriving at a receiver position. As such, it is a parameter representing the attenuation properties of a medium in a single backscattering model and was originally studied to evaluate the attenuation of coda waves from small local earthquakes^27,28^. It is dimensionless, frequency-dependent, and can be expressed as in Eq. (2), which is valid for both the single scattering and diffusion theories. The amplitude of an ultrasonic signal $A(\omega |t)$ at a frequency around $\omega$ over a time *t* is expressed by a source term *c*, which is constant for a uniform excitation. The constant *a* accounts for geometrical spreading, where $a=1$ is generally assumed for body waves.

2 $$\begin{aligned} A(\omega |t) = ct^{-a}e^{-{\omega }t/{2Q}} \end{aligned}$$

As we use embedded sensors, we can assume that the ultrasonic coda waves are mainly energy transported by body waves. We can rewrite the equation by replacing $\omega$ with the frequency *f*, around which the signal is filtered.

3 $$\begin{aligned} A(f|t) = ct^{-1}e^{-{\pi }ft/Q} \end{aligned}$$

By transforming the equation, the right side of Eq. (4) can be interpreted as a linear function with a negative slope of $\pi f/Q$. It must be noted that the quality factor Q is inversely proportional to attenuation.

4 $$\begin{aligned} \ln {[A(f|t)t]} = \ln {c}-\frac{{\pi }f}{Q}t \end{aligned}$$

**Figure 1:**
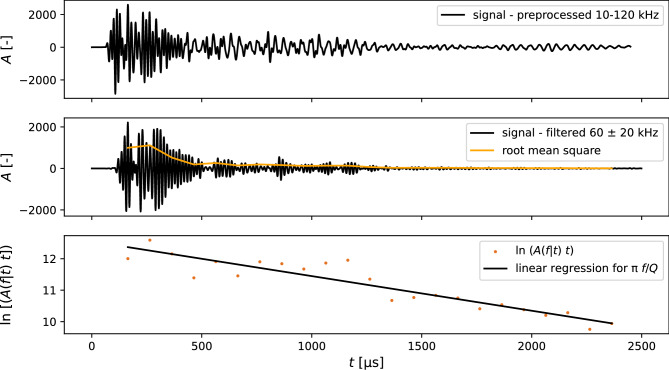
Procedure for deriving the quality factor *Q* using the signal’s root mean square^34^. Starting from the preprocessed signal, the signal is further filtered around a central frequency *f*, e.g., 60 kHz with a bandwidth of  ±20 kHz. An approximation for *Q* according to Eq. (4) is the slope of the linear regression using the root mean square of the signal amplitude *A* for a constant *f*.

The procedure for deriving *Q* is illustrated in Fig. 1 for an ultrasonic signal, where the root mean square of the amplitude is the basis for linear regression, starting from the maximum amplitude of the filtered signal. The computation of *Q* according to Eq. (2) is affected by reflections at geometrical boundaries that can be observed in the form of periodic wave packages in the root mean square in Fig. 1. *Q* is lapse-time dependent^33^, which we do not explore here. Therefore, our absolute estimate of *Q* is rather uncertain and we focus on the relative measurements.

### Materials

We produced two batches of concrete specimens with constant cement content and aggregate size distributions (Table 1). Only the water-cement ratio was varied, inducing an increased pore space volume within the cement paste matrix for an increasing water-to-cement ratio^35^. The cement used in both mixes was Ordinary Portland Cement *CEM I 42.5 R* and the aggregates were primarily quartzitic and crushed. To decrease the viscosity of the fresh concrete and enhance the coupling of the embedded sensors, we added 2.4 % of polycarboxylatether-based superplasticizer to the concrete with $w/c=0.45$. A standardized pressure method test yielded an air void content of 2.0 % air voids for $w/c=0.45$ and 0.8 % air voids for $w/c=0.60$ in the fresh concrete.

After production, the specimens were covered with water-retaining jute mats for 24 h and further cured under water for more than 100 d. The levels of chemical and autogenous shrinkage were minimal due to the high degree of hydration^36,37^ and thus negligible for the subsequent experiment. However, drying shrinkage along with carbonation shrinkage^38,39^ are significant while decreasing the relative humidity as part of the experimental setup and are discussed later in the article.

**Table 1:** Raw materials and their composition for the two concrete mixes with $w/c = 0.45$ and $w/c = 0.60$, where the aggregates are primarily quartzitic and crushed. The aggregates also contain 1.2 % oversized grains 16.0/22.4 mm. The cement type is CEM I 42.5 R, and the superplasticizer (as mass fraction in % of the cement content) is *BASF MasterEase 3880*.

Cement content [kg/m 3]	Water-cement ratio [-]	Aggregate size [mm] and their ratio [%]				Superplasticizer
		0/2	2/5	5/8	8/16	
350	0.45	40	12	29	19	2.4
350	0.60	40	12	29	19	0

### Experimental setup

**Figure 2:**
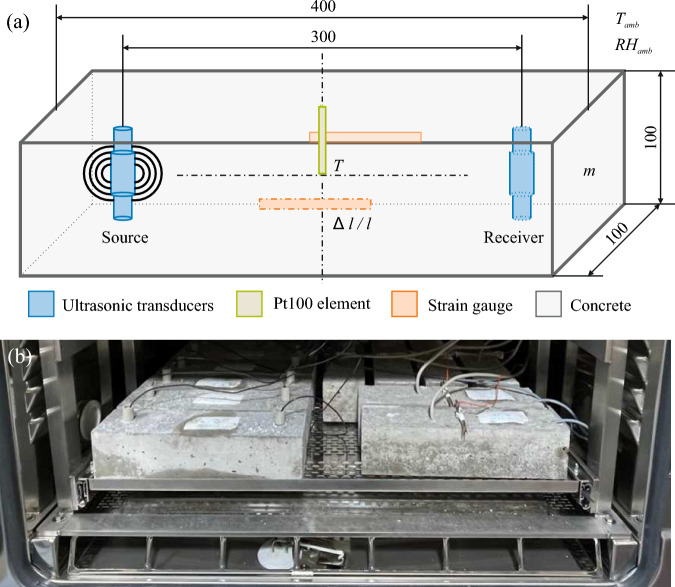
(**a**) The geometry of the specimens for all conducted investigations is a cuboid with an edge length of 400 mm and a square cross-section of 100 x 100 mm^2^ For both concrete mixes ($w/c=[0.45,0.60]$), three specimens are equipped with ultrasonic transducers and another three specimens with a Pt100 element (*T*). The specimens’ mass *m* is monitored for three further specimens. (**b**) Photograph of the experimental setup in the climate chamber at controlled ambient temperature $T_{amb}$ and relative humidity $RH_{amb}$.

To investigate the impact of both temperature and relative humidity on the ultrasonic coda, we varied the ambient parameters temperature $T_{amb}$ and relative humidity $RH_{amb}$ while monitoring the concrete specimens’ internal condition using embedded sensors and mass tracking. The set of ambient and embedded sensors, together with the geometrical setup, is presented in Fig. 2a. It consisted of ambient temperature and relative humidity sensors in the climate chamber and an embedded Pt100 element for temperature *T* [$^\circ$C] measurement positioned at the center of each specimen. Strain gauges (*PL-60-11-3LJCT-F*) with a gauge resistance of $R=120\,\Omega$ and a length of $l=60\,mm$, applied to the surface on two opposite sides and implemented as a quarter bridge, tracked the strain. In addition, the mass *m* of specimens from the same batch was continuously tracked. Three specimens were monitored for each *T*, *m*, and the ultrasonic signals. The ultrasonic signals were retrieved from symmetrically positioned embedded transducers, each at a distance of 300 mm from the center axis (Fig. 2a), which act as sender-receiver pairs with a center frequency of 62 kHz^40^, proposed for CWI in concrete structures^41^. The measurement system (see^42^ for further details) continuously samples signals with a length of 12,000 samples at a rate of 1 MHz and a 14 bit resolution. Fig. 2b shows the experimental setup of concrete specimens with embedded sensors in the climate chamber. Additional specimens without any measurement equipment are regularly weighed to track changes in mass,i.e., water loss. Each specimen set for the ultrasonic measurements, the temperature and strain measurements, and for the mass tracking contained three specimens.

The experimental variations in temperature and relative humidity had to be chosen carefully because several mechanisms other than the non-destructive boundaries trigger damage in the concrete matrix, thus contributing to superimposed changes in the ultrasonic velocity variations.Several processes can introduce damage on a microstructural level due to thermal loading^43^. These can be physicochemical processes, i.e., the decomposition of hydrates^44^, by microchemically induced cracking in the interfacial transition zone between the aggregates and the cement paste due to differential dilation^45^, by drying shrinkage due to differential strains^46^, by increasing vapor pressure^47^ or by their gradients^48^. When elevating the temperature, ettringite is the first hydrate phase to decompose. It is a crystalline thermally unstable component of Portland cement, and the decomposition of its phase-pure form is discussed in the literature for combinations of temperature and vapor pressure^49^. We varied the temperature between 55 °C and 2 $^\circ$C, while the relative humidity was modulated between 95 % RH and 35 % RH avoiding any ettringite decomposition. Further decomposition mechanisms beyond the stated temperatures and relative humidity do not apply because the study’s objective is to determine the relations in the non-destructive regime.

**Figure 3:**
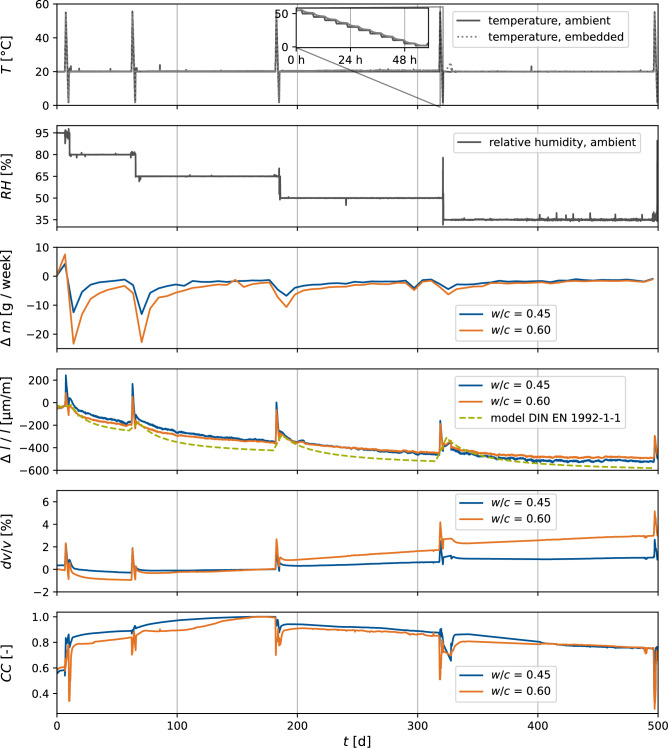
Experimental measurements with the ambient and embedded control variables *T* and *RH*. The mass losses $\Delta m$ for both specimen series $w/c=0.45$ and $w/c=0.60$ were monitored weekly, and daily immediately after each temperature variation cycle. Strains $\Delta l/l$ were monitored throughout the experiment based on the mean of readings taken from two strain gauges (quarter bridges) and compared to a model for drying shrinkage according to *DIN EN 1992-1-1*^50^. Throughout the experiment, the velocity variations *dv*/*v* were computed based on Eq. (1).

Figure 3 presents the modulation of the external control variables $T_{amb}$ and $RH_{amb}$ in the climate chamber together with the specimens’ mass changes $\Delta m$, where the starting conditions are 95 % RH at 20 $^\circ$C. The relative humidity is held constant at a temperature of 20 $^\circ$C until the mass change decreases below a threshold of 0.05 % per week. After reaching a nearly constant mass, we assumed a constant water saturation in the concrete matrix and introduced a stepwise temperature variation starting at 55 $^\circ$C. We decreased the temperature in 5 $^\circ$C steps with a last step of 3 $^\circ$C down to a minimum of 2 $^\circ$C. Each temperature was held for a 5 h duration to condition the entire specimen at the same temperature, validated by equalization of the temperature of the embedded central sensor with the ambient temperature. After the temperature variation, the relative humidity was decreased by 15 % until the final step which reached 35 % RH. The monitored strain matched the predicted strain from the model according to *DIN EN 1992-1-1*^50^. We conclude that drying shrinkage is responsible for the measured strain in our experiment.

## Results

### Temperature impact on the coda

**Figure 4:**
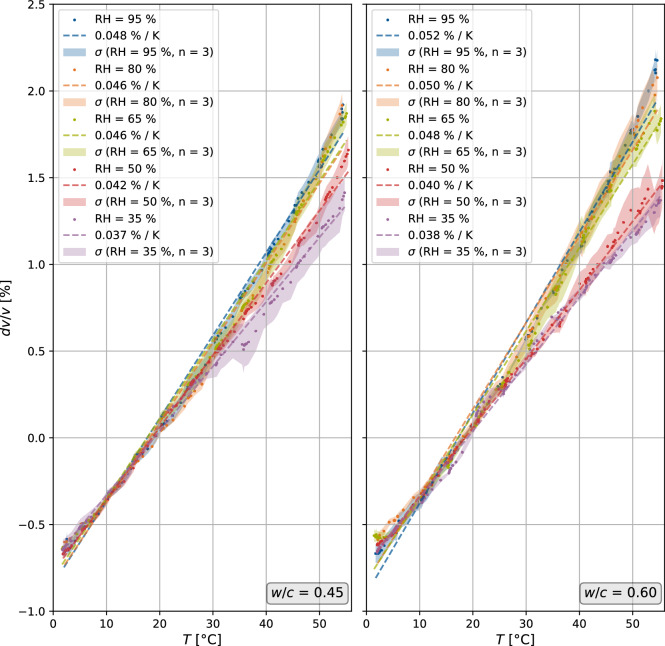
Velocity variation *dv*/*v* of the ultrasonic signals plotted against temperature for the two concrete mixes with $w/c=0.45$ and $w/c=0.60$. Dots represent the data points, whereas the dashed line is a linear regression for each data set. The $R^2$ values are all greater than 0.985 for all mixes and states. The transparent fill shows the standard deviation $\sigma$ for all three specimens. The reference signals are selected at a temperature *T* of approximately $20\,^{\circ }$C.

Ultrasonic measurements were conducted as the temperature was varied between 55 °C and 2 $^{\circ }$C once the specimens had reached a constant relative humidity state, which was validated by their steady mass. The results for each relative humidity state are plotted in Fig. 4 for two concrete mixes with different *w*/*c* ratios. Their standard deviations, $\sigma$, and linear regressions are also shown. The slopes of the regression are indicated as the velocity variation per Kelvin, while the reference signal, used to normalize the velocity variation values, was selected at 20 $^{\circ }$C for each temperature variation cycle.

For both investigated concrete mixes, the slope of the velocity variation plots steadily decreases as the relative humidity decreases, because the degree of saturation is lower. However, we note a larger gap between maximum and minimum slopes for the mix with $w/c=0.60$. The difference between the two mixes is particularly notable at higher saturation levels. As the mixes only differ in the ratio *w*/*c*, we can derive an interdependency between the change in the slope and the concrete’s pore system. The pore volume, as well as the portion of the water stored in it, and the average pore size, are larger for $w/c=0.60$. For this concrete, more water evacuates from the pore system in between the temperature cycles due to more dynamic water transport processes, thus resulting in an increase in the slope.

### Relative humidity impact on the coda

**Figure 5:**
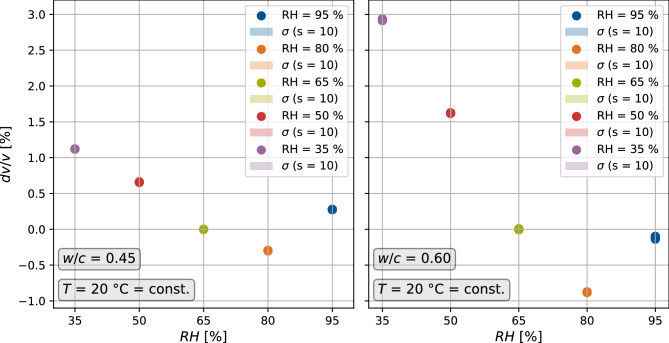
Velocity variation *dv*/*v* of the ultrasonic signals plotted against relative humidity for the two concrete mixes with $w/c=0.45$ and $w/c=0.60$. Dots represent the data points, where the standard deviations for the ten signals collected at half-hourly intervals are smaller than the dots in this figure.

Only constant-state ultrasonic signals, validated by a nearly constant mass, were evaluated to derive the relationship between the velocity variations and the relative humidity of the specimens. We intentionally ignored the transition times between humidity regimes in order to ignore fluid diffusion processes and focus on steady-state regimes. Figure 5 shows, for both concrete mixes, the correlation between *dv*/*v* and *RH* at a constant temperature $T=20\,^{\circ }$C for ten signals immediately before each temperature variation cycle as depicted in Fig. 3.

For both mixes, the reference signals, used to normalize the velocity variation values, were chosen at 65 % RH. Figure 5 shows similar trends for both mixes: *dv*/*v* decreases with increasing concrete relative humidity until a value of 80 % is reached. The value at 95 % RH is an exception for both mixes because in this state, almost all pores are filled and a further reduction of *dv*/*v* is expected when compared to the state at 80 % RH. Once again, the mix with $w/c=0.60$ exhibits larger differences between the *RH* states, which we interpret as a consequence of a higher pore volume and more extensive transport processes.

### Combined impact of temperature and relative humidity

The results from Figs. 4 and 5 with variations of only one impact variable form the basis for investigations on the combined effect of temperature and relative humidity on the coda for a variable *w*/*c* ratio. Data from both sets were matched for $T=20\,^\circ C$, where we defined a boundary condition in Eq. (5).

5 $$\begin{aligned} dv/v\;(T=20\,^\circ C\,|\,RH=65\,\%)=0 \end{aligned}$$

Using polynomial regression, the measurement data were fitted to the following third-order polynomial over three variables, the water-to-cement ratio *w*/*c*, the temperature *T*, and relative humidity *RH* (Eq. (6)). This fitting procedure uses the data for compensation when analyzing ultrasonic measurements and evaluating them under varying ambient conditions.

6 $$\begin{aligned} dv/v = \sum ^{i=3}_{i=0} \sum ^{j=3}_{j=0} \sum ^{k=3}_{k=0} a_{ijk} \left( \frac{w}{c} \right) ^{i}T^{j}RH^{k} \end{aligned}$$

**Figure 6:**
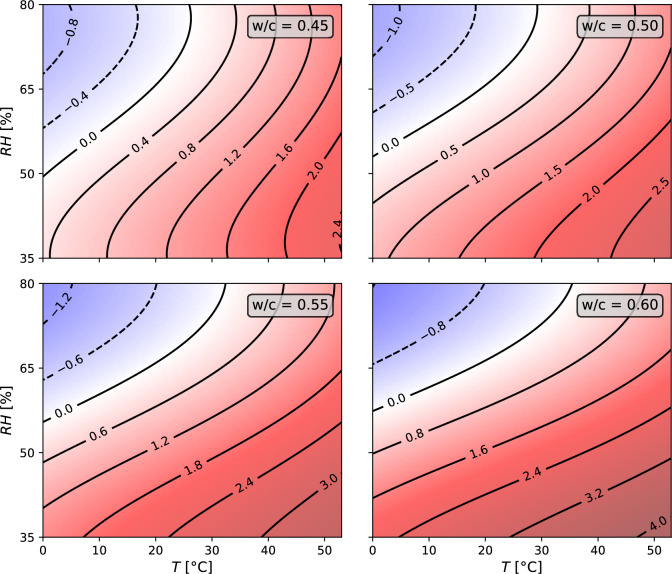
Contour plots of the velocity variation *dv*/*v* for four water-to-cement ratios dependent on temperature and relative humidity. The plots for $w/c=0.45$ and $w/c=0.60$ are based on measurements, while the plots for $w/c=0.50$ and $w/c=0.55$ are predictions of the third-order polynomial model. The lines in the plots are iso-lines of constant relative velocity variation.

In the above equation, $a_{ijk}$ refers to the polynomial coefficients that were fitted to the measurement data. The corresponding values are provided in Table 2 in the Appendix. The predictions of the model for values of the temperature, relative humidity, and water-to-cement ratios for the whole range of values within which the measurement data is available are shown in Fig. 6.

We provide the metrics of the model fit, where the $R^2$ value is 0.999, and the mean-squared error is $1.5604\times 10^{-7}$.

The above polynomial equation was obtained using the ordinary least squares method by performing linear regression on the polynomial features. The sklearn python package was used for performing the regression. To identify the most sensitive components of the polynomial, the dataset was standardized using the mean $\mu$ and the standard deviation $\sigma$ in Eq. (7).

7 $$\begin{aligned} x'=\frac{x-\mu (x)}{\sigma \left( x\right) } \end{aligned}$$

The following expression (Eq. (8)) for the relative velocity change ignores terms that are one order of magnitude smaller than the largest coefficient. The relative velocity change is most sensitive to the relative humidity, then the temperature, and finally, the water-to-cement ratio.

8 $$\begin{aligned} \left( \frac{dv}{v}\right) '\propto 0.26T'-0.50RH'-0.36RH' \left( \frac{w}{c}\right) '+(0.26T'-0.49RH') {\left( \frac{w}{c} \right) '}^{2}+0.14RH'^{3} \end{aligned}$$

### Impact on the quality factor

**Figure 7:**
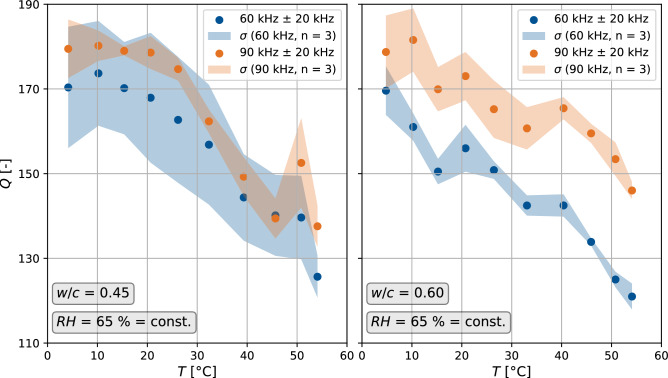
Quality factor *Q* for temperature variations for the two concrete mixes with $w/c = 0.45$ and $w/c = 0.60$, each preconditioned at $RH=65\,\%$. *Q* was evaluated for two central frequencies 60 kHz and 90 kHz.

The quality factor *Q* was computed based on the procedure in Fig. 1 using Eq. (2). First, *Q* was evaluated as a function of *T* for three specimens at two central frequencies (Fig. 7): 60 kHz, which is approximately the resonance frequency of the sensor, and 90 kHz, which is still considered to be in the simple scattering regime of concrete^41^. Both signal filters had a bandwidth of 20 kHz around the central frequency.

For both concrete mixes, *Q* generally decreased for higher temperatures. Thus, the signal attenuation increased for both frequencies with increasing temperature. *Q* was reduced by about 40 between 10 °C and 50 $^\circ$C for both compositions. If the absolute values of *Q* are not interpretable due to multiple wave reflections in the specimen, the interpretation of the changes in *Q* should still be reliable.

**Figure 8:**
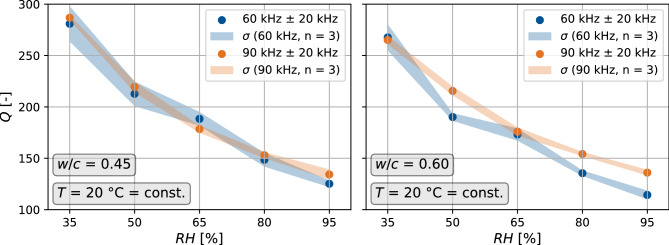
Quality factor *Q* for relative humidity variations for the two concrete mixes with $w/c = 0.45$ and $w/c = 0.60$ at a constant temperature of 20 $^{\circ }$C. *Q* was evaluated for two center frequencies 60 kHz and 90 kHz.

Additionally, we obtain a similar, continuous, decreasing trend in *Q* for increasing relative humidity for both frequencies and both mixes. The more saturated the pore space, the higher is the attenuation of the ultrasonic signals (Fig. 8). *Q* has a significantly higher spread in values than when the temperature is varied: values of *Q* differ around 115 % over the measured humidity range. Therefore, the sensitivity of attenuation to relative humidity is much stronger than the temperature sensitivity.

To show the sensitivity of the attenuation properties to *T*,*RH*, and *w*/*c*, we provide a standardized polynomial expression using the computed quality factors in the identical parameter space as used in Eq. (8). For both increasing temperature and relative humidity, we identify a negative correlation with *Q*, thus, increasing attenuation.

9 $$\begin{aligned} Q\propto (-0.18T'-0.38RH')(1 + \left( \frac{w}{c}\right) '^{2})+0.14T\cdot RH'-0.11T'^{2}+0.18RH'^{2} \end{aligned}$$

Despite the dependence in *Q* with lag time, frequency, and window length, this relation is the first to suggest a coupling of the temperature and humidity effects in affecting attenuation. Recent work also shows the negative correlation between *Q* and *T* and water content in sandstone^51^. Their correlations exhibit strong dependence on the pore space of the sample, which is suggested and formulated by our regression.

## Discussion

We evaluated non-destructive and reversible state changes in concrete using CWI, a technique characterized by high precision and sensitivity. Further, we evaluated the monitoring of anelastic properties in ultrasonic attenuation. We explored these states by changing the most commonly varying environmental factors, namely temperature and relative humidity. We found that temperature profoundly influenced ultrasonic speed and relative humidity on attenuation.

Where changes in ultrasonic velocity *dv*/*v* due to temperature are concerned, our results are comparable with previous studies to within an order of magnitude^8,52^, although the values for the regression slopes (Fig. 4) are slightly smaller in our study. The relationship between temperature and *dv*/*v* is quasi-linear for the same water-to-cement ratio, and simple linear regressions all show high $R^2$-values, all above 0.985, confirming an accurate fit to the experimental data.

In our experimental design, we ensured a homogeneous temperature distribution over the entire volume of the specimens with embedded sensors and therefore avoided the influence of gradients in the material. Thanks to the relative humidity control of the climate chamber during the temperature variation and the relatively short duration of the temperature cycles, slightly more than two days, we minimized the effect of drying at the geometrical edges, which otherwise amplifies a hysteresis between cooling and heating cycles^52^. The impact of near-edge drying on *dv*/*v* is difficult to quantify and decreases for larger concrete sample geometries and greater distances between the structural edges and the sensors, where at least the first factor is generally the case for real civil engineering structures. We also evaluated the impact of relative humidity variations on the material. We increased the experiment time significantly due to the slow diffusion transport processes in the concrete’s pore system. We validated a relative humidity distribution that was sufficiently constant to meet our requirements by measuring changes in the mass of specimens, which is a precise and reliable measurement method. Nevertheless, we assume minor relative humidity gradients in the material decrease from the core. These gradients, however, still exist after years of conditioning, and we consider the introduced mass change threshold as a valid criterion for a nearly constant relative humidity distribution. In addition, we compared the theoretical drying shrinkage (negative volumetric strains) based on *DIN EN1992-1-1* with our strain measurements in Fig. 3. It could be demonstrated that the measured strain was approximately equal to the predicted strain, and no further substantial strain change was expected for each state. We consider that the separation of the impact of strain on *dv*/*v*, induced by drying shrinkage, and the pure impact of water saturation of the concrete’s pore system, induced by varying relative humidity, is unnecessary because the two effects always occur simultaneously.

Interpreting the results in Fig. 5, we find a steady decrease of *dv*/*v* for an increase of water in the pore system up to 80 % RH. This trend is disrupted for the highest saturation at 95 % RH, where *dv*/*v* increases compared to the previous state. Additionally, the evaluation of the quality factor *Q* yielded a steady increase in attenuation for all investigated states as relative humidity increases (Fig. 8).

In addition, the ultrasonic signal correlation between 65 % RH and 95 % RH shows a relatively small *CC* of approximately 0.6. In contrast, all other signals below 80 % RH as used in plotting Fig. 5, have a $CC>0.8$. Investigating the ultrasonic waveforms, we found a strong attenuation of the shear wave for high saturation, which we explain as due to the presence of fully saturated pores. As coda waves are dominated by S waves^53^, the strong attenuation may induce significant waveform distortion such that the stretching technique suffers from cycle skipping. Figure 3 shows a jump in the *CC* values between 95 % RH and 80 % RH that is not observed for the later step-decrease in relative humidity. CWI often measures confidence using *CC* values. As a result of this high uncertainty in interpreting the *dv*/*v* measurements at such high saturation, we excluded the 95 % RH state from the polynomial model in Fig. 6.

Typically in seismological research, the contributions of the temperature and relative humidity effects are summed in a linear combination to explain *dv*/*v*^17,54^, though these relations have strong spatial heterogeneity^19,20^. Here we noticed a change in sensitivity to temperature depending on the relative humidity. Hence, we recast the problem using a polynomial regression model to perform linear regression on the polynomial features. The polynomial features are the model variables, i.e., the water-to-cement ratio, the temperature, relative humidity, and their powers up to a polynomial degree of 3. Due to the specificity of the experimental setup, we are not interpreting the absolute values of the coefficients. We propose a way to solve for relative humidity and temperature given the two ultrasonic observables *dv*/*v* and *Q*. Furthermore, we can interpret that the relations are not strictly linear if one compares materials with different porous properties.

As the model is an analytical model, an analysis of the sensitivity of the relative velocity change to the ambient conditions and the water-to-cement ratio can be performed. The model can readily compensate for ambient conditions when performing damage detection in concrete structures under non-standard weather conditions. It should be noted that the model cannot be extrapolated and must only be used within the limits of the experimental measurements, especially since we noted a strong sensitivity of *dv*/*v* to large *RH* values.

As an alternative to the approach used here, we tested a Gaussian Process Regression and found a good fit. However, the lack of interpretability hindered the characterization of the physical processes.

## Conclusions

The objective of our study was to derive relationships between the ambient variables temperature and relative humidity, and changes in ultrasonic signals in concrete evaluated using Coda wave interferometry and attenuation measurements. Thanks to our experimental design, we were first able to establish one-dimensional relationships for the sole variation of either temperature or relative humidity.

We confirmed the findings of previous studies^8,52^, which demonstrated an approximately linear effect of temperature variation on the velocity variation of ultrasonic signals. Furthermore, we extended these investigations to include the impact of relative humidity variations. We were able to show that the stretching method is an appropriate and sensitive method for measuring the velocity variation of the signals for state changes between 30 % RH and 80 % RH. However, our measurements only had a low confidence level based on the signal correlation coefficient for the state at 95 % RH, where the concrete’s pore system is highly saturated with water. We hypothesized that the signal perturbations were too large and caused decreasing confidence in CWI for the highly saturated state.

We substantiated this hypothesis by introducing the quality factor to study the signal attenuation, which is particularly sensitive to microstructure and its damage. Its evaluation showed a decreasing value of the quality factor (e.g., an increase in attenuation) for a higher degree of relative humidity. The steady trend for the analysis of *Q* finally reinforced the presumed low confidence for the particular data point at very high saturation.

As we observe changes in both temperature and relative humidity in natural settings, we extended our investigations with a polynomial model that accounts for the simultaneous variation of both variables. Furthermore, we embedded the water-to-cement ratio into our model, which defines the characteristics of the concrete pore system. For a higher *w*/*c* ratio, we expect a greater pore volume and, therefore, faster dynamics regarding the fluid transport processes in the material. We could confirm this assumption by explicitly analyzing the velocity variation of the ultrasonic signal, which showed greater sensitivity to temperature and relative humidity changes for a greater water-to-cement ratio (i.e., greater pore space). Considering all three variables leads to our model being of the third order.

Evaluating the changes in phase and amplitude of ultrasonic signals in concrete allows the detection of even weak changes induced by ambient conditions. To subsequently detect irreversible changes or damage in the framework of an early-warning system, we conclude that the proportion of velocity and attenuation variation changes, generated by ambient variables, can be characterized and hence compensated using our modeling approach^55^. For the separation of temperature-induced changes, we consider the simultaneous monitoring of *T* using embedded sensors to be the most elegant way of controlling its impact on *dv*/*v*, while the observation over a period that covers the typical temperature range, may be sufficient for deriving the *dv*/*v*(*T*) relationship because of its linearity. Regarding the impact of the relative humidity on *dv*/*v*, the change is slow and only visible over a long-term perspective. However, changes in *Q* are significant, and attenuation may be used instead of *dv*/*v* to compensate for relative humidity. Therefore, we propose an ambient ultrasonic field approach to monitoring the internal state of concrete that will provide a more accurate assessment if or when structural damage occurs.

## Data Availability

The datasets used and/or analysed during the current study available from the corresponding author on reasonable request.

## A Polynomial model coefficients

See Table 2.

**Table 2:** List of coefficients of the third order polynomial obtained by polynomial regression.

*n*	*a*[*ijk*]	*i*	*j*	*k*
0	$-$1.235967e-01	0	0	0
1	9.621853e-02	1	0	0
2	3.631449e-04	0	1	0
3	5.107886e-03	0	0	1
4	1.344006e-01	2	0	0
5	1.380768e-04	1	1	0
6	$-$2.651369e-03	1	0	1
7	$-$1.546802e-06	0	2	0
8	$-$2.261435e-06	0	1	1
9	$-$7.619478e-05	0	0	2
10	1.151416e-01	3	0	0
11	4.693153e-05	2	1	0
12	$-$4.163067e-03	2	0	1
13	$-$7.605707e-06	1	2	0
14	5.858475e-06	1	1	1
15	2.860905e-05	1	0	2
16	1.809416e-08	0	3	0
17	9.979734e-08	0	2	1
18	$-$3.073448e-08	0	1	2
19	3.765474e-07	0	0	3
